# Serum Galectin-3 Levels Correlate with Reduced Vascular Reactivity in Patients with Coronary Artery Disease

**DOI:** 10.3390/medicina62061018

**Published:** 2026-05-24

**Authors:** Po-Yu Huang, Min-Han Hsieh, Ji-Hung Wang, Jen-Pi Tsai, Bang-Gee Hsu

**Affiliations:** 1 Division of Nephrology, Department of Internal Medicine, Dalin Tzu Chi Hospital, Buddhist Tzu Chi Medical Foundation, Chiayi 62247, Taiwan; 2 Institute of Medical Sciences, Tzu Chi University, Hualien 97004, Taiwan; 3 Department of Internal Medicine, Hualien Tzu Chi Hospital, Buddhist Tzu Chi Medical Foundation, Hualien 97004, Taiwan; 4 School of Medicine, Tzu Chi University, Hualien 97004, Taiwan; 5 Division of Cardiology, Hualien Tzu Chi Hospital, Buddhist Tzu Chi Medical Foundation, Hualien 97004, Taiwan; 6 Division of Nephrology, Hualien Tzu Chi Hospital, Buddhist Tzu Chi Medical Foundation, Hualien 97004, Taiwan

**Keywords:** galectin-3, endothelial dysfunction, vascular reactivity index, coronary artery disease

## Abstract

*Background and Objectives*: Endothelial dysfunction is essential in the development and progression of coronary artery disease (CAD) and its complications. Galectin-3 mediates inflammation and organ fibrosis and promotes endothelial dysfunction. Meanwhile, the vascular reactivity index (VRI) reflects endothelial function. The purpose of this research was to evaluate the association between serum galectin-3 levels and VRI in patients diagnosed with CAD. *Materials and Methods*: One hundred and eighteen patients with CAD were enrolled. Endothelial function was noninvasively evaluated using digital thermal monitoring, and VRIs were obtained. According to VRI values, patients were classified into good (≥2.0), intermediate (1.0–1.9), and poor (<1.0) subgroups. Galectin-3 levels were quantified using an enzyme-linked immunosorbent assay. *Results*: Patients with poor vascular reactivity were older in age (*p* = 0.028) and had higher serum total cholesterol (*p* = 0.003), low-density lipoprotein cholesterol (*p* = 0.005), and galectin-3 (*p* < 0.001) levels. Multivariable stepwise linear regression analysis revealed galectin-3 as an independently associated factor of lower VRIs (β = −0.488; *p* < 0.001). Logistic regression model confirmed that galectin-3 independently was associated with higher odds of vascular reactivity dysfunction (odds ratio, 1.120; 95% confidence interval, 1.016–1.235; *p* = 0.023) or poor vascular reactivity (odds ratio, 1.445; 95% confidence interval, 1.179–1.772; *p* < 0.001). *Conclusions*: Serum galectin-3 is independently associated with reduced VRIs and endothelial dysfunction in patients with CAD.

## 1. Introduction

Coronary artery disease (CAD) is characterized by atherosclerotic lesions within the coronary arteries, with genetic and environmental factors predisposing patients to CAD [1]. Cardiovascular events are the second-most common cause of fatality in 2019 [2]. In addition to traditional risk factors such as high blood pressure and high plasma glucose, endothelial dysfunction, inflammation, oxidative stress, and vascular calcification contribute to atherosclerotic cardiovascular diseases [3,4]. The endothelium is a crucial component in maintaining homeostasis of the coronary vasculature by mediating vascular tone, inflammatory response, coagulation, and permeability [5]. Both traditional and nontraditional cardiovascular risk factors can result in endothelial dysfunction, thereby accelerating the formation of atherosclerotic plaques [6]. Several methods are used to evaluate the vascular endothelium [7]. Using the digital thermal monitoring (DTM)-derived vascular reactivity index (VRI), peripheral artery tonometry noninvasively assesses the degree of reactive hyperemia after vessel occlusion, which is a surrogate marker of microvascular endothelial function [8,9]. Evaluation of endothelial function assists clinicians in determining cardiovascular risk and the long-term prognosis of patients [10,11]. Specifically, atherosclerosis-associated disorders, intima–media thickness of the coronary arteries, major adverse cardiovascular events, need for revascularization, and heart failure can all be potentially predicted by the status of coronary or peripheral endothelial function assessed invasively or non-invasively.

Galectin-3, which is a member of the β-galactoside-binding galectin family, contributes to inflammation and vital organ fibrosis [12]. Additionally, it is involved in the development of atherosclerosis and alteration of endothelial function through multiple pathways [13]. Histologically, galectin-3 molecules are generated by macrophages and have been identified within atherosclerotic plaques. Increased galectin-3 expression gives rise to macrophage infiltration within the plaques and predisposes these plaques to structural progression and instability [14,15]. Either higher baseline levels of or interval increase in serum galectin-3 potentially acts as a biomarker to predict adverse cardiovascular outcomes, including heart failure rehospitalization, left ventricular remodeling, cardiac fibrogenesis, and cardiovascular deaths [16,17]. Notably, elevated serum galectin-3 levels are independently correlated with poorer vascular reactivity in patients with chronic kidney disease and hypertension. These studies also discovered that C-reactive protein, a critical cardiovascular risk variable, is positively correlated with galectin-3 blood levels. Additionally, among hypertensive patients with concomitant cardiovascular events, the long-term survival is significantly lower, provided that they have higher galectin-3 blood levels [18,19].

The association between serum galectin-3 and parameters of endothelial function, based on DTM-derived VRI, in patients with CAD is unknown. Therefore, this study investigated the association between serum galectin-3 levels and the severity of endothelial dysfunction among patients with underlying CAD.

## 2. Materials and Methods

### 2.1. Enrolled Patients and Anthropometric Measurements

Overall, 118 patients with stable underlying CAD who received regular outpatient care from a general hospital in Hualian, Taiwan, were enrolled from January 2021 through July 2021. The diagnosis of CAD was made when coronary angiography showed at least a fifty-percent intraluminal stenosis of one or more epicardial coronary arteries [20]. Each participant was requested to provide their informed consent before study enrollment. The exclusion criteria included participants with active infectious diseases, unstable angina, acute myocardial infarction, malignancy, limb amputation, liver cirrhosis, stroke, chronic obstructive pulmonary disease, uncontrolled arrhythmia, and volume overload with acute pulmonary edema. The study protocol was approved in advance by the Research Ethics Committee of Hualian Tzu Chi Hospital, Buddhist Tzu Chi Medical Foundation (Project No. IRB108-219-A).

Data regarding age, gender, and medication regimen (specifically angiotensin-converting enzyme inhibitors, angiotensin receptor blockers, beta blockers, calcium channel blockers, statins, and fibrates) were retrieved from electronic medical records. In accordance with practice guidelines, hypertension was diagnosed if the systolic blood pressure (SBP) was ≥140 mm Hg, the diastolic blood pressure (DBP) was ≥90 mm Hg, and/or participants were taking antihypertensive agents [21]. Based on the diagnostic criteria of the American Diabetes Association, diabetes mellitus (DM) was documented when hemoglobin A1C equals or exceeds 6.5%, fasting plasma glucose equals or exceeds 126 mg/dL, random plasma glucose equals or exceeds 200 mg/dL, or when participants were taking antidiabetic medications [22].

Body height and weight measurements were performed after the patients fasted for at least eight hours. The results of the height and weight were rounded off to the nearest half centimeters and half kilograms, respectively. Body mass index (BMI) was calculated using the following formula: weight (kg)/height^2^ (m^2^).

### 2.2. Biochemical Testing from Blood Sampling

For each participant, an eight-hour fasting blood sample (approximately 5 mL) was taken on the day of the outpatient clinic visit. These blood specimens were promptly centrifuged at 3000× *g* for 10 min. The serum was isolated after centrifugation, stored at 4 °C, and processed for measurement within sixty minutes. Serum levels of fasting glucose, albumin, blood urea nitrogen (BUN), creatinine, total cholesterol (TCH), triglycerides, high-density lipoprotein cholesterol (HDL-C), and low-density lipoprotein cholesterol (LDL-C) were measured using an automated analyzer (Advia 1800; Siemens Healthcare, Erlangen, Germany). Serum galectin-3 levels were measured using commercial enzyme-linked immunosorbent assay kits (RayBiotech, Georgia, GA, USA) [23]. We used the 2021 Chronic Kidney Disease Epidemiology Collaboration equation to output the estimated glomerular filtration rate (eGFR) of each participant.

### 2.3. Estimation of the Endothelial Function

Participants were advised to avoid smoking, alcohol, and caffeine ingestion throughout the night prior to endothelial function testing. We also recommended against the use of agents affecting vasomotor tone. A noninvasive DTM (VENDYS-II; Endothelix, Inc., Houston, TX, USA) operated by the same investigator was used to evaluate endothelial function. Patients were instructed to maintain a supine position for 30 min in a thermostatic space with a temperature of 22–24 °C. Temperature sensors were attached to the distal parts of both index fingers, and the results were surrogate markers of vascular function. The complete procedure was divided into three sections. First, baseline temperatures of the tips of the index fingers were measured through a sensor for 5 min. Second, a cuff used for blood pressure measurement was inflated over the right upper arm for occlusion of the vessels for up to 5 min. The third step involved immediate cuff deflation, and the rebound fingertip temperature was detected after 5 min of the hyperemic phase. VRI scores were calculated using VENDYS software. Based on the VRI results, the vascular reactivity was categorized as good (VRI ≥ 2.0), intermediate (VRI 1.0–1.9), and poor (VRI < 1.0) [24].

### 2.4. Statistical Analysis

The Shapiro–Wilk method was used to check the presence of normal distribution of the individual data. Continuous variables are presented as means ± standard deviation or median with interquartile range, whereas categorical variables are presented as counts and percentages. Comparisons among VRI categories were done using one-way analysis of variance for normally distributed variables and the Jonckheere–Terpstra test for non-normally distributed variables (comprising body height, fasting glucose, triglyceride, BUN, and creatinine in this study). Categorical variables were analyzed using the Cochran–Armitage test for trend analysis. Before the regression analysis, variables lacking normal distribution were log-transformed to achieve normality. Simple linear regression was performed to determine variables associated with VRI; we then adopted the significant parameters into the multivariable forward stepwise regression analysis. To reduce the risk of model overfitting, candidate variables entered into the multivariable models were restricted to those significantly associated with VRI in univariable analyses. In addition to the forward stepwise regression model, a forced-entry multivariable linear regression model was also performed as a sensitivity analysis. Multicollinearity was assessed using the variance inflation factor. We evaluated the relationship between serum galectin-3 levels and the other variables using Spearman’s rank correlation coefficient. Univariable and multivariable logistic regression analyses were performed to evaluate the association between galectin-3 levels and vascular reactivity dysfunction (intermediate or poor) and poor vascular reactivity alone. The area under the receiver operating characteristic (ROC) curves was calculated to evaluate the discriminatory ability of galectin-3 levels for identifying vascular reactivity dysfunction and poor vascular reactivity (MedCalc v22.019, Ostend, Belgium). Statistical significance was set at a *p*-value of 0.05. The statistical analytical processes were performed using SPSS Statistics for Windows, version 19.0 (IBM Corp., Armonk, NY, USA).

## 3. Results

Table 1 presents the baseline demographic information and clinical features of the participants. Of the 118 participants, 85.5% were men. Additionally, 54 (45.8%) had DM, and 62 (52.5%) had underlying hypertensive disorders. Based on their VRI, 50 (42.4%), 56 (47.5%), and 12 (10.1%) participants had good, intermediate, and poor vascular reactivity, respectively. Advanced age (*p* = 0.028) and higher serum concentrations of TCH (*p* = 0.003), LDL-C (*p* = 0.005), and galectin-3 (*p* < 0.001) were significantly associated with impaired vascular reactivity. By contrast, no significant differences were detected with respect to body height or weight, BMI, SBP or DBP, triglyceride, HDL-C, fasting glycemic level, albumin, BUN, creatinine, eGFR, sex, DM, hypertension, or use of blood pressure-lowering and lipid-lowering agents among the groups.

Simple linear regression analysis revealed that younger age (*r* = −0.232; *p* = 0.011), lower serum TCH (*r* = −0.233; *p* = 0.011), lower LDL-C (*r* = −0.226; *p* = 0.014), and lower galectin-3 levels (*r* = −0.488; *p* < 0.001) were significantly associated with higher VRIs (Table 2). Further multivariable stepwise linear regression analysis, adopting the significant variables in simple linear regression analysis (age, TCH, LDL-C, and galectin 3), identified only higher serum galectin-3 concentrations (β = −0.488; adjusted R^2^ change = 0.231; *p* < 0.001) as a factor independently associated with decreased VRI in patients with CAD. In the forced-entry multivariable linear regression model including age, TCH, LDL-C, and galectin-3, serum galectin-3 remained independently and negatively associated with VRI (β = −0.448, *p* < 0.001). All variance inflation factor values were below 5, indicating no severe multicollinearity among the included variables.

Spearman’s rank correlation coefficient measured the clinical correlates of serum galectin-3. Older age (ρ = 0.480; *p* < 0.001), higher TCH levels (ρ = 0.198; *p* = 0.032), and higher LDL-C levels (ρ = 0.187; *p* = 0.043) were significantly correlated to higher galectin-3 levels. Additionally, low VRI values (ρ = −0.488; *p* < 0.001), hypoalbuminemia (ρ = −0.266; *p* = 0.004), and reduced eGFR (ρ = −0.334; *p* < 0.001) were significantly associated with higher galectin-3 levels (Table 3). These parameters were considered potential risk factors for elevated serum galectin-3 levels. On the contrary, BMI, SBP, DBP, log-triglyceride, HDL-C, and log-glucose were not significantly associated with galectin-3 concentrations in these patients.

Vascular reactivity dysfunction encompasses both intermediate and poor vascular reactivity. We then analyzed the association between galectin-3, vascular reactivity dysfunction, and poor vascular reactivity using logistic regression (Table 4). After adjusting for parameters significantly associated with vascular reactivity dysfunction and poor vascular reactivity in the crude model (i.e., age, TCH, and LDL-C), multivariable logistic regression analysis revealed that higher serum galectin-3 levels were independently associated with higher odds of vascular reactivity dysfunction (odds ratio, 1.120 for each 1 ng/mL increment in serum galectin-3 level; 95% confidence interval [CI], 1.016–1.235; *p* = 0.023) as well as poor vascular reactivity (odds ratio, 1.445 for each 1 ng/mL increment in serum galectin-3 level; 95% CI, 1.179–1.772; *p* < 0.001).

The area under the ROC curve analysis revealed that galectin-3 could distinguish vascular reactivity dysfunction from good vascular reactivity (area under the curve, 0.648; 95% CI, 0.547–0.748; *p* = 0.004). Additionally, galectin-3 showed good discriminatory ability for identifying poor vascular reactivity (area under the curve, 0.913; 95% CI, 0.816–1.000; *p* < 0.001). Based on the Youden index, the ideal cutoff value of galectin-3 for predicting vascular reactivity dysfunction was 10.62 ng/mL, yielding a sensitivity of 75.0%, specificity of 54.0%, positive predictive value of 68.9%, and negative predictive value of 61.4%. Regarding the poor vascular reactivity, the ideal cutoff was 15.73 ng/mL, yielding a sensitivity of 91.7%, specificity of 85.9%, positive predictive value of 42.3%, and negative predictive value of 98.9% (Table 5).

## 4. Discussion

The main finding is that serum galectin-3 levels are independently negatively associated with the VRI in patients with CAD. Increased serum galectin-3 levels were independently associated with vascular reactivity dysfunction and poor vascular reactivity in patients diagnosed with CAD, and serum galectin-3 levels were positively associated with age, TCH, and LDL-C and negatively correlated with albumin and eGFR.

Endothelial dysfunction is a key driver of coronary arteriopathy. The molecular mechanisms underlying endothelial dysfunction are complex, with diminished nitric oxide availability, aberrant generation of reactive oxygen species, and activation of inflammatory cascades being involved [25]. Notably, oxidized LDLs may be an important mediator of endothelial dysfunction as they induce the formation of atherosclerotic plaques by promoting endothelial cell senescence, converting endothelial cells into a mesenchymal phenotype, recruiting leukocytes, and promoting adhesion and epigenetic modifications [26]. The DTM-derived VRI is a reliable method for estimating microcirculatory function [27]. Vascular reactivity is associated with the degree of coronary artery calcifications and Framingham risk scores [28]. Nevertheless, whether VRI possesses prognostic value in cardiovascular diseases remains unclear [29].

A remarkable result of this study is the identification of serum galectin-3 levels as being independently associated with VRI values; the area under the ROC curve of galectin-3 in differentiating poor vascular reactivity from relatively preserved vascular reactivity was up to 0.913. These findings suggest that serum galectin-3 levels may reflect endothelial function status in patients with CAD. Galectin-3 induces endothelial dysfunction by mediating inflammatory responses and enhancing oxidative stress [30]. Galectin-3 overexpression within atherosclerotic plaques is also associated with increased chemokine production and monocyte chemotaxis [31]. In contrast, downregulated galectin-3 protein expression resulted in decreased proinflammatory cytokine production in human dendritic cells [32]. Regarding oxidative stress, galectin-3 acts synergistically with oxidized LDL to amplify reactive oxygen species generation and reduce antioxidant activity in cultured human umbilical vein endothelial cells [33]. In an obese mouse model, galectin-3 genetic deletion decreased reactive oxygen species production that was induced by nicotinamide adenine dinucleotide phosphate oxidase 1, thereby improving endothelial dysfunction and vascular tone [34]. Meanwhile, hypoalbuminemia and low eGFR were significantly associated with higher galectin-3 levels. Low serum albumin levels is a biomarker of an inflammatory state [35]. In chronic kidney disease, which is a proinflammatory state, oxidative stress, cellular hypoxia, and decreased endothelial glycocalyx content increase the vulnerability to endothelial dysfunction [36]. Therefore, galectin-3 is an essential factor in the pathophysiology of endothelial dysfunction and atherogenesis.

Based on the VRI, dyslipidemia is also associated with endothelial dysfunction. Oxidized LDLs are essential in the progression of endothelial dysfunction. Small-dense LDLs, which comprise a fraction of the total LDL-C, also exhibit atherogenic properties by promoting endothelial dysfunction and foam cell infiltration [37]. Collectively, dyslipidemia is closely associated with atherosclerosis and arteriopathy-related sequelae [38]. Additionally, blood LDL-C concentrations correlated with flow-mediated vasodilation among patients without statin treatment [39]. The association between advancing age and poor VRIs was also confirmed in our study. As age increases, endothelial nitric oxide synthase uncoupling occurs, and the content of the endothelial glycocalyx decreases, predisposing elderly patients to endothelial dysfunction and vasculopathy [40].

The functional and pathophysiologic roles of galectin-3 in CAD and other atherosclerotic diseases have been explored in other clinical studies. One research study enrolled patients with acute coronary syndrome and chronic coronary syndrome. Serum galectin-3 concentrations were significantly associated with a higher risk of the occurrence of acute coronary syndrome as well as greater severity of intraluminal stenosis and thicker plaques of atherosclerotic vessels. The optimal cutoff of galectin-3 for differentiating acute coronary syndrome from chronic coronary syndrome was 20.18 ng/mL [41]. Another study evaluated the clinical correlates of blood galectin-3 among hospitalized patients who were diagnosed with myocardial infarction and who received coronary revascularization. Galectin-3 levels higher than 8.7 ng/mL could predict overall deaths in post-myocardial infarction patients. Galectin-3 levels were significantly associated with the number of critically stenotic coronary vessels. The research also discovered that elevated galectin-3 was significantly correlated with older age, impaired kidney function, and increased intima–media thickness of the coronary artery [42]. Some of these associations were consistent with our study findings. In fact, these parameters are closely linked to the burden of endothelial malfunction. This study differs from previous research that primarily focused on acute coronary syndrome, post-myocardial infarction prognosis, or the severity of coronary stenosis. Instead, our study specifically focuses on the association between serum galectin-3 levels and DTM-derived VRI in patients with stable CAD.

This study has important clinical implications. We can conveniently and indirectly assess the severity of endothelial dysfunction by measuring galectin-3 levels in patients with CAD, allowing for the early identification and management of modifiable cardiovascular risk factors. Such diagnostic processes and interventions may improve patients’ overall health and clinical outcomes. Furthermore, dyslipidemia is a well-established and modifiable risk factor for endothelial dysfunction, and lipid-lowering therapies that reduce serum TCH and LDL-C levels can prevent further worsening of vascular impairment.

Some strengths of the present study can be highlighted. The result of the area under the ROC curves demonstrated the excellent capability of galectin-3 in identifying poor endothelial function in CAD patients. Furthermore, we utilized various statistical methods to delineate certain risk factors of elevation in galectin-3 levels, which helps better understand the pathogenic role of galectin-3 in endothelial dysfunction.

There are a few limitations in this study. First, this study had a cross-sectional design; thus, we could only determine the association rather than the causal relationship between galectin-3 and VRI. Future experiments with longitudinal designs are mandatory. Second, this was a single-center study with a restricted sample size, which might limit the generalizability of the results. Third, we did not weigh other inflammatory biomarkers, including C-reactive protein and interleukins; therefore, we were unable to clearly explain the putative inflammatory mechanisms underlying galectin-3-mediated endothelial function. Moreover, we did not measure the levels of oxidized LDL or small-dense LDL, which might better reflect the burden of endothelial dysfunction.

## 5. Conclusions

In patients with underlying CAD, serum galectin-3 levels are independently associated with endothelial dysfunction as reflected by the DTM-derived VRI. These findings suggest that galectin-3 may serve as a biomarker associated with impaired endothelial function in patients with CAD. However, given the cross-sectional design of this study, future longitudinal studies are needed to clarify whether galectin-3 is associated with subsequent cardiovascular outcomes or changes in endothelial function over time. Additionally, future preclinical and clinical studies may further investigate whether galectin-3-targeted strategies could attenuate endothelial dysfunction in patients with atherosclerotic disease.

## Figures and Tables

**Table 1:** Comparison of clinical characteristics across vascular reactivity index categories in 118 patients with coronary artery disease.

Characteristics	All Participants (*n* = 118)	Good Vascular Reactivity (*n* = 50)	Intermediate Vascular Reactivity (n = 56)	Poor Vascular Reactivity (n = 12)	*p* Value for Trend
Age (years)	63.12 ± 8.96	61.35 ± 8.38	63.46 ± 9.33	68.90 ± 7.35	0.028 *
Height (cm)	167.00 (160.38–169.25)	166.50 (159.75–169.25)	167.00 (161.25–169.75)	165.50 (157.00–169.50)	0.489
Body weight (kg)	71.97 ± 11.99	70.81 ± 11.00	73.41 ± 13.07	70.12 ± 10.76	0.462
Body mass index (kg/m^2^)	26.42 ± 3.76	26.19 ± 3.65	26.68 ± 3.98	26.19 ± 3.31	0.787
Vascular reactivity index	1.87 ± 0.65	2.45 ± 0.31	1.65 ± 0.22	0.51 ± 0.25	<0.001 *
Systolic BP (mmHg)	131.51 ± 18.24	133.84 ± 17.56	129.11 ± 17.52	133.00 ± 23.94	0.396
Diastolic BP (mmHg)	76.64 ± 13.20	77.90 ± 15.32	76.11 ± 11.38	73.83 ± 11.94	0.584
Total cholesterol (mg/dL)	162.66 ± 33.90	156.89 ± 29.93	162.07 ± 29.18	191.77 ± 51.39	0.003 *
Triglyceride (mg/dL)	126.50 (95.00–181.50)	116.50 (94.75–193.50)	126.50 (91.75–167.25)	157.50 (109.25–207.50)	0.541
HDL-C (mg/dL)	46.47 ± 10.88	48.06 ± 11.81	45.32 ± 10.53	45.25 ± 8.04	0.401
LDL-C (mg/dL)	93.52 ± 29.60	92.52 ± 26.18	88.98 ± 26.24	118.83 ± 44.92	0.005 *
Fasting glucose (mg/dL)	107.00 (91.00–136.75)	109.00 (94.75–141.25)	106.00 (90.25–134.75)	106.00 (91.50–137.75)	0.669
Albumin (g/dL)	4.38 ± 0.24	4.41 ± 0.27	4.37 ± 0.19	4.26 ± 0.27	0.133
Blood urea nitrogen (mg/dL)	17.00 (14.00–20.00)	16.00 (14.75–18.25)	17.00 (14.00–21.00)	17.00 (13.25–24.25)	0.742
Creatinine (mg/dL)	1.00 (0.80–1.10)	1.00 (0.80–1.10)	1.00 (0.80–1.10)	1.00 (0.90–1.35)	0.560
eGFR (mL/min)	82.83 ± 24.13	84.33 ± 25.95	83.32 ± 22.80	74.30 ± 22.46	0.428
Galectin-3 (ng/mL)	12.81 ± 5.09	11.24 ± 3.81	12.38 ± 4.20	21.35 ± 5.56	<0.001 *
Male, *n* (%)	100 (85.5)	41 (82.0)	48 (87.5)	11 (91.7)	0.607
Diabetes mellitus, *n* (%)	54 (45.8)	25 (50.0)	24 (42.9)	5 (41.7)	0.729
Hypertension, *n* (%)	62 (52.5)	28 (56.0)	30 (53.6)	4 (33.3)	0.361
ACE inhibitor use, *n* (%)	23 (19.5)	11 (22.0)	11 (19.0)	1 (8.3)	0.562
ARB use, *n* (%)	51 (43.2)	24 (48.0)	23 (41.1)	4 (33.3)	0.592
β-blocker use, *n* (%)	60 (50.8)	22 (44.0)	31 (55.4)	7 (58.3)	0.435
CCB use, *n* (%)	47 (39.8)	22 (44.0)	19 (33.9)	6 (50.0)	0.429
Statin use, *n* (%)	99 (83.9)	40 (80.0)	49 (87.5)	10 (83.3)	0.576
Fibrate use, *n* (%)	9 (7.6)	4 (8.0)	4 (7.1)	1 (8.3)	0.982

Data are presented as mean ± standard deviation for continuous variables with a normal distribution, as median (interquartile range) for continuous variables without a normal distribution, and as number (%) for qualitative variables. Group differences were assessed using one-way analysis of variance, the Jonckheere–Terpstra test, and the Cochran–Armitage trend test, respectively. BP, blood pressure; HDL-C, high-density lipoprotein cholesterol; LDL-C, low-density lipoprotein cholesterol; eGFR, estimated glomerular filtration rate; ACE, angiotensin-converting enzyme; ARB, angiotensin receptor blocker; CCB, calcium channel blocker. * *p* < 0.05 was considered statistically significant.

**Table 2:** Correlation of vascular reactivity indices and clinical variables by simple or multivariable linear regression analyses among 118 coronary artery disease patients.

Variables	Vascular Reactivity Index				
	Simple Regression		Multivariable Regression		
	*r*	*p* Value	Beta	Adjusted *R*^2^ Change	*p* Value
Age (years)	−0.232	0.011 *	–	–	–
Log-Height (cm)	−0.035	0.705	–	–	–
Body weight (kg)	−0.081	0.386	–	–	–
Body mass index (kg/m^2^)	−0.064	0.494	–	–	–
Systolic blood pressure (mmHg)	0.090	0.331	–	–	–
Diastolic blood pressure (mmHg)	0.153	0.099	–	–	–
Total cholesterol (mg/dL)	−0.233	0.011 *	–	–	–
Log-Triglyceride (mg/dL)	−0.033	0.720	–	–	–
HDL-C (mg/dL)	0.106	0.255	–	–	–
LDL-C (mg/dL)	−0.226	0.014 *	–	–	–
Log-Glucose (mg/dL)	0.153	0.097	–	–	–
Albumin (g/dL)	0.151	0.104	–	–	–
Log-BUN (mg/dL)	−0.054	0.559	–	–	–
Log-Creatinine (mg/dL)	−0.126	0.175	–	–	–
eGFR (mL/min)	0.117	0.209	–	–	–
Galectin-3 (ng/mL)	−0.488	<0.001 *	−0.488	0.231	<0.001 *

Because of skewed distributions, height, triglyceride, fasting glucose, blood urea nitrogen, and creatinine were log-transformed before analysis. Simple linear regression and multivariable stepwise linear regression analyses were used to determine factors associated with vascular reactivity index, with adjustment for age, total cholesterol, LDL-C, and galectin-3 in the multivariable model. HDL-C, high-density lipoprotein cholesterol; LDL-C, low-density lipoprotein cholesterol; BUN, blood urea nitrogen; eGFR, estimated glomerular filtration rate. * *p* < 0.05 was considered statistically significant.

**Table 3:** Spearman correlation coefficients between serum galectin-3 and the other variables in 118 coronary artery disease patients.

Variables	Spearman Coefficient of Correlation	*p* Value
Age (years)	0.480	<0.001 *
Body mass index (kg/m^2^)	−0.169	0.067
Vascular reactivity index	−0.488	<0.001 *
Systolic blood pressure (mmHg)	0.054	0.563
Diastolic blood pressure (mmHg)	−0.075	0.422
Total cholesterol (mg/dL)	0.198	0.032 *
Log-Triglyceride (mg/dL)	−0.044	0.635
HDL-C (mg/dL)	−0.061	0.509
LDL-C (mg/dL)	0.187	0.043 *
Log-Glucose (mg/dL)	−0.005	0.956
Albumin (g/dL)	−0.266	0.004 *
eGFR (mL/min)	−0.334	<0.001*

Because triglyceride and glucose levels were not normally distributed, these variables were logarithmically transformed before analysis. Associations were evaluated using Spearman’s rank correlation analysis. HDL-C, high-density lipoprotein cholesterol; LDL-C, low-density lipoprotein cholesterol; eGFR, estimated glomerular filtration rate. * *p* < 0.05 was considered statistically significant (two-tailed).

**Table 4:** Univariable and multivariable logistic regression analysis for vascular reactivity dysfunction (intermediate vascular reactivity and poor vascular reactivity) or poor vascular reactivity among 118 coronary artery disease patients.

Model	Galectin-3 (Per 1 ng/mL of Increase) for Vascular Reactivity Dysfunction		Galectin-3 (Per 1 ng/mL of Increase) for Poor Vascular Reactivity	
	OR (95% CI)	*p* Value	OR (95% CI)	*p* Value
Crude model	1.131 (1.037–1.234)	0.006 *	1.483 (1.231–1.787)	<0.001 *
Model 1	1.120 (1.016–1.235)	0.023 *	1.445 (1.179–1.772)	<0.001 *

Adjusted model: age, total cholesterol, and low-density lipoprotein cholesterol. OR, odds ratio; CI, confidence interval. * *p* < 0.05 was considered statistically significant.

**Table 5:** Discriminatory performance of galectin-3 for identifying vascular reactivity dysfunction (intermediate vascular reactivity and poor vascular reactivity) or poor vascular reactivity.

	**Vascular Reactivity** **Dysfunction**						
	**AUC (95% CI)**	***p* Value**	**Cut-Off**	**Sen (%)**	**Spe (%)**	**PPV (%)**	**NPV (%)**
Galectin-3 (ng/mL)	0.648 (0.547–0.748)	0.004 *	10.62	75.0	54.0	68.9	61.4
	**Poor Vascular Reactivity**						
	**AUC (95% CI)**	***p* Value**	**Cut-Off**	**Sen (%)**	**Spe (%)**	**PPV (%)**	**NPV (%)**
Galectin-3 (ng/mL)	0.913 (0.816–1.000)	*p* < 0.001 *	15.73	91.7	85.9	42.3	98.9

Abbreviations: AUC, area under the curve; 95% CI, 95% confidence interval; Sen, sensitivity; Spe, specificity; PPV, positive predictive value; NPV, negative predictive value. * *p* < 0.05 was considered statistically significant.

## Data Availability

The author responsible for correspondence is able to provide the data employed in this research upon request.

## Abbreviations

The following abbreviations are used in this manuscript:

BMI	Body mass index
BUN	Blood urea nitrogen
CAD	Coronary artery disease
DBP	Diastolic blood pressure
DM	Diabetes mellitus
DTM	Digital thermal monitoring
eGFR	Estimate glomerular filtration rate
LDL-C	Low-density lipoprotein cholesterol
ROC	Receiver operating characteristic curve
SBP	Systolic blood pressure
TCH	Total cholesterol
TG	Triglyceride
VRI	Vascular reactivity index

## References

[1] Agrawal H., Choy H.K., Liu J., Auyoung M., Albert M.A. (2020). Coronary artery disease. Biol. Arterioscler. Thromb. Vasc. Biol..

[2] Ueng K.C., Chiang C.E., Chao T.H., Wu Y.W., Lee W.L., Li Y.H., Ting K.H., Su C.H., Lin H.J., Su T.C. (2023). 2023 guidelines of the Taiwan society of cardiology on the diagnosis and management of chronic coronary syndrome. Acta Cardiol. Sin..

[3] Perone F., Bernardi M., Spadafora L., Betti M., Cacciatore S., Saia F., Fogacci F., Jaiswal V., Asher E., Paneni F. (2025). Non-traditional cardiovascular risk factors: Tailored assessment and clinical implications. J. Cardiovasc. Dev. Dis..

[4] Wang L., Lei J., Wang R., Li K. (2023). Non-traditional risk factors as contributors to cardiovascular disease. Rev. Cardiovasc. Med..

[5] Józkowiak M., Niebora J., Domagała D., Data K., Partyńska A., Kulus M., Kotrych K., Podralska M., Górska A., Chwiłkowska A. (2025). New insights into endothelial cell physiology and pathophysiology. Biomed. Pharmacother..

[6] Widlansky M.E., Gokce N., Keaney J.F., Vita J.A. (2003). The clinical implications of endothelial dysfunction. J. Am. Coll. Cardiol..

[7] Chia P.Y., Teo A., Yeo T.W. (2020). Overview of the assessment of endothelial function in humans. Front. Med..

[8] Ahmadi N., McQuilkin G.L., Akhtar M.W., Hajsadeghi F., Kleis S.J., Hecht H., Naghavi M., Budoff M. (2011). Reproducibility and variability of digital thermal monitoring of vascular reactivity. Clin. Physiol. Funct. Imaging.

[9] Bockus L., Kim F. (2022). Coronary endothelial dysfunction: From pathogenesis to clinical implications. Open Heart.

[10] Flammer A.J., Anderson T., Celermajer D.S., Creager M.A., Deanfield J., Ganz P., Hamburg N.M., Lüscher T.F., Shechter M., Taddei S. (2012). The assessment of endothelial function: From research into clinical practice. Circulation.

[11] Minhas A.S., Goerlich E., Corretti M.C., Arbab-Zadeh A., Kelle S., Leucker T., Lerman A., Hays A.G. (2022). Imaging assessment of endothelial function: An index of cardiovascular health. Front. Cardiovasc. Med..

[12] Bouffette S., Botez I., De Ceuninck F. (2023). Targeting galectin-3 in inflammatory and fibrotic diseases. Trends Pharmacol. Sci..

[13] Gao Z., Liu Z., Wang R., Zheng Y., Li H., Yang L. (2020). Galectin-3 is a potential mediator for atherosclerosis. J. Immunol. Res..

[14] Colombo P.B., Camargo J.V.S., Campo V.L., Marchiori M.F., Ribeiro A.B. (2026). The role of galectin-3 in atherosclerosis and its cardiovascular complications: An update. J. Lipid Atheroscler..

[15] Varasteh Z., De Rose F., Mohanta S., Li Y., Zhang X., Miritsch B., Scafetta G., Yin C., Sager H.B., Glasl S. (2021). Imaging atherosclerotic plaques by targeting galectin-3 and activated macrophages using ((89)Zr)-DFO- galectin3-F(ab’)(2) mAb. Theranostics.

[16] Dong R., Zhang M., Hu Q., Zheng S., Soh A., Zheng Y., Yuan H. (2018). Galectin-3 as a novel biomarker for disease diagnosis and a target for therapy (Review). Int. J. Mol. Med..

[17] Madrigal-Matute J., Lindholt J.S., Fernandez-Garcia C.E., Benito-Martin A., Burillo E., Zalba G., Beloqui O., Llamas-Granda P., Ortiz A., Egido J. (2014). Galectin-3, a biomarker linking oxidative stress and inflammation with the clinical outcomes of patients with atherothrombosis. J. Am. Heart Assoc..

[18] Hsu B.G., Wang C.H., Lai Y.H., Tsai J.P. (2021). Serum galectin-3 level is positively associated with endothelial dysfunction in patients with chronic kidney disease stage 3 to 5. Toxins.

[19] Wang H.S., Hsu B.G., Wang J.H., Yang C.F. (2024). Increased serum galectin-3 level is associated with endothelial dysfunction and cardiovascular events in patients with hypertension. Heliyon.

[20] Meijboom W.B., Van Mieghem C.A., van Pelt N., Weustink A., Pugliese F., Mollet N.R., Boersma E., Regar E., van Geuns R.J., de Jaegere P.J. (2008). Comprehensive assessment of coronary artery stenoses: Computed tomography coronary angiography versus conventional coronary angiography and correlation with fractional flow reserve in patients with stable angina. J. Am. Coll. Cardiol..

[21] Weber M.A. (2014). Recently published hypertension guidelines of the JNC 8 panelists, the American Society of Hypertension/International Society of Hypertension and other major organizations: Introduction to a focus issue of the Journal of Clinical Hypertension. J. Clin. Hypertens. (Greenwich).

[22] American Diabetes Association Professional Practice Committee for Diabetes (2026). 2. Diagnosis and classification of diabetes: Standards of care in diabetes-2026. Diabetes Care.

[23] Huang P.Y., Huang C.S., Lin Y.L., Chen Y.H., Hung S.C., Tsai J.P., Hsu B.G. (2023). Positive association of serum galectin-3 with the development of aortic stiffness of patients on peritoneal dialysis. J. Clin. Med..

[24] Lo W.L., Hsu B.G., Wang C.H., Lin Y.L., Kuo C.H., Lai Y.H. (2025). Lipoprotein(a) levels predict endothelial dysfunction in maintenance hemodialysis patients: Evidence from vascular reactivity index assessment. Ren. Fail..

[25] Wang X., He B. (2024). Endothelial dysfunction: Molecular mechanisms and clinical implications. MedComm.

[26] Jiang H., Zhou Y., Nabavi S.M., Sahebkar A., Little P.J., Xu S., Weng J., Ge J. (2022). Mechanisms of oxidized LDL-mediated endothelial dysfunction and its consequences for the development of atherosclerosis. Front. Cardiovasc. Med..

[27] Naghavi M., Yen A.A., Lin A.W., Tanaka H., Kleis S. (2016). New indices of endothelial function measured by digital thermal monitoring of vascular reactivity: Data from 6084 patients registry. Int. J. Vasc. Med..

[28] Ahmadi N., Hajsadeghi F., Gul K., Vane J., Usman N., Flores F., Nasir K., Hecht H., Naghavi M., Budoff M. (2008). Relations between digital thermal monitoring of vascular function, the Framingham risk score, and coronary artery calcium score. J. Cardiovasc. Comput. Tomogr..

[29] Minson C.T., Green D.J. (2008). Green, measures of vascular reactivity: Prognostic crystal ball or Pandora’s box?. J. Appl. Physiol..

[30] Seropian I.M., Cassaglia P., Miksztowicz V., González G.E. (2023). Unraveling the role of galectin-3 in cardiac pathology and physiology. Front. Physiol..

[31] Papaspyridonos M., McNeill E., de Bono J.P., Smith A., Burnand K.G., Channon K.M., Greaves D.R. (2008). Galectin-3 is an amplifier of inflammation in atherosclerotic plaque progression through macrophage activation and monocyte chemoattraction. Arterioscler. Thromb. Vasc. Biol..

[32] Chen S.S., Sun L.W., Brickner H., Sun P.Q. (2015). Downregulating galectin-3 inhibits proinflammatory cytokine production by human monocyte-derived dendritic cells via RNA interference. Cell. Immunol..

[33] Ou H.C., Chou W.C., Hung C.H., Chu P.M., Hsieh P.L., Chan S.H., Tsai K.L. (2019). Galectin-3 aggravates ox-LDL-induced endothelial dysfunction through LOX-1 mediated signaling pathway. Environ. Toxicol..

[34] Padgett C.A., Bátori R.K., Speese A.C., Rosewater C.L., Bush W.B., Derella C.C., Haigh S.B., Sellers H.G., Corley Z.L., West M.A. (2023). Galectin-3 mediates vascular dysfunction in obesity by regulating NADPH oxidase 1. Arterioscler. Thromb. Vasc. Biol..

[35] Allison S.P., Lobo D.N. (2024). The clinical significance of hypoalbuminaemia. Clin. Nutr..

[36] Wang J.H., Lin Y.L., Hsu B.G. (2025). Endothelial dysfunction in chronic kidney disease: Mechanisms, biomarkers, diagnostics, and therapeutic strategies. Tzu Chi Med. J..

[37] Bekbossynova M., Saliev T., Ivanova-Razumova T., Andossova S., Kali A., Myrzakhmetova G. (2025). Small dense LDL: An underestimated driver of atherosclerosis (Review). Mol. Med. Rep..

[38] Higashi Y. (2023). Endothelial function in dyslipidemia: Roles of LDL-cholesterol, HDL-cholesterol and triglycerides. Cells.

[39] Matsui S., Kajikawa M., Hida E., Maruhashi T., Iwamoto Y., Iwamoto A., Oda N., Kishimoto S., Hidaka T., Kihara Y. (2017). Optimal target level of low-density lipoprotein cholesterol for vascular function in statin naïve individuals. Sci. Rep..

[40] Donato A.J., Machin D.R., Lesniewski L.A. (2018). Lesniewski, Mechanisms of dysfunction in the aging vasculature and role in age-related disease. Circ. Res..

[41] Onea H.L., Homorodean C., Lazar F.L., Negrea M.O., Calin T., Bitea I.C., Teodoru M., Nechita V.I., Olteanu A.L., Olinic D.-M. (2025). Galectin-3 reflects systemic atherosclerosis in patients with coronary artery disease. Medicina.

[42] Lisowska A., Knapp M., Tycińska A., Motybel E., Kamiński K., Święcki P., Musiał W.J., Dymicka-Piekarska V. (2016). Predictive value of Galectin-3 for the occurrence of coronary artery disease and prognosis after myocardial infarction and its association with carotid IMT values in these patients: A mid-term prospective cohort study. Atherosclerosis.

